# Evolutionary conformation model of salivary gland lithiasis

**DOI:** 10.3389/froh.2025.1610977

**Published:** 2025-06-05

**Authors:** Álvaro Sánchez Barrueco, María Victoria López-Acevedo Cornejo, William Aragonés Sanzen-Baker, Sol López-Andrés, Gonzalo Díaz Tapia, Ignacio Alcalá Rueda, Jessica Mireya Santillán Coello, Carlos Cenjor Español, José Miguel Villacampa Aubá

**Affiliations:** ^1^ Medicine Faculty, Alfonso X el Sabio University, Madrid, Spain; ^2^ ENT and Cervicofacial Surgery Department, Fundación Jiménez Díaz University Hospital, Madrid, Spain; ^3^ Department of Medicine, Villalba General University Hospital, Collado Villalba, Spain; ^4^ Department of Mineralogy and Petrology, Faculty of Geological Sciences, Universidad Complutense, Madrid, Spain; ^5^ Department of Medicine, Universidad Autónoma de Madrid, Madrid, Spain

**Keywords:** sialolithiasis, biomineralization, heterogeneous nucleation, sialomicroliths, conformational model, sialadenitis, scanning electron microscopy

## Abstract

**Introduction:**

Salivary stones, or sialoliths, are calcified concretions forming within salivary glands and their ducts through a two-stage process: an initial formation of a central core via precipitation of inorganic material mediated by organic substances, followed by layering of additional organic and inorganic material. Substrates for sialolith formation include mucoid agglomerates, organic vesicles, foreign bodies, and bacterial biofilms. Understanding the detailed structure of sialoliths may aid in developing specific preventive or therapeutic strategies.

**Materials and methods:**

This study analyzed 137 sialoliths from 102 patients treated across three university hospitals. Stones were extracted via sialendoscopy, direct extraction, or spontaneous extrusion. Structural and compositional analyses were conducted using scanning electron microscopy (SEM-EDX) and x-ray diffraction (XRD).

**Results:**

Most sialoliths were from the submandibular gland (82%), with the remainder from the parotid gland (18%). Parotid stones predominantly exhibited irregular shapes, while submandibular stones were generally ellipsoidal. All stones demonstrated an oolitic structure characterized by a central core surrounded by concentric layers and frequently associated with bacteria. Mineral composition predominantly included octacalcium phosphate (OCP), hydroxyapatite, and whitlockite. Larger sialoliths exhibited a higher proportion of hydroxyapatite, indicating increased crystallinity compared to OCP.

**Discussion:**

Despite diverse origins and locations, sialoliths share common morphological and compositional traits. Their formation begins with heterogeneous nucleation of calcium phosphates around organic spherules, likely induced by bacterial biofilms. These initial nuclei aggregate into a central core upon which additional layers of organic and inorganic materials deposit progressively. This layering increases the size and crystallinity of the sialoliths over time. The coexistence of amorphous phases and structural heterogeneity within layers explains the variability among stones. Detailed SEM-EDX analysis supports a unified conformational model for sialoliths that integrates the interplay of organic substrates, inorganic minerals, bacterial biofilms, and temporal factors.

**Conclusions:**

Sialoliths are oolitic aggregates featuring a central core surrounded by concentric layers composed of organic and inorganic materials. Their formation process involves initial heterogeneous nucleation, bacterial influence, and progressive crystallization. This universal conformational model effectively describes sialolith formation irrespective of patient-specific or anatomical variations.

## Introduction

Salivary stones, also known as sialoliths, are calcified concretions that form within the salivary ducts, including the main duct, hilum, and intraglandular ducts. These biominerals, like those formed in geological environments, possess morphological and compositional characteristics that reveal their developmental history and the processes they have experienced. Regarding the ontogeny of these calcifications, the most general theories suggest that their formation occurs in two stages. The first stage involves the creation of a central core through the precipitation of inorganic salts mediated by certain organic substances. The second stage consists of the accumulation of layers of organic and inorganic material on this core. Factors such as pH variation, reduced salivary flow, and increased calcium concentration are considered to decrease the solubility of calcium phosphates in saliva, contributing to their precipitation (1, 2).

The literature proposes three possible substrates for the formation of the initial core: (a) floating mucoid materials in the Wharton duct (generically referred to as a mucus plug), (b) vesicles filled with organic matter or lipid droplets (3) that give rise to so-called sialomicroliths, and (c) a foreign body that penetrates retrogradely from the oral cavity (2). The resulting macroscopic core appears to be composed of an organoid matrix mixed with exosomatic vesicles, loose calcium apatite crystals, and bacteria (4).

Other authors suggest that sialomicroliths form physiologically in the autophagosomes of the salivary glands. These are asymptomatically released into the salivary lumen. Occasionally, these sialomicroliths can impact and cause local micro-obstruction, leading to chronic focal sialadenitis (5–7) that exacerbates the release of mucoid material from the duct walls. Various studies have linked the invasion of oral germs into the major salivary duct, either parotid or submandibular, with the genesis of lithiasis (4, 8). RNA studies through polymerase chain reaction or microscopy have found colonization by germs, particularly the *Streptococcus genus*, whose species are part of the oral microbiome (9, 10). These germs were found as isolated bacterial cells and/or bacterial aggregates within colonies, surrounded by amorphous glycoprotein material with a laminar appearance, suggesting biofilm organization.

The core thus formed increases in size by the apposition of organic and inorganic layers, progressively constituting the sialolith. Recent works focused on the genesis of renal stones, consider these lithiasis as the result of overlapping multiple processes, including medical, biological, and geological factors (11).

The aim of this study is to conduct a detailed analysis of the morphologies, textures, structures, zonations, chemical composition, and mineralogy of salivary stones to understand the mechanisms of their formation and growth, identify possible predisposing factors, and develop effective therapeutic strategies to prevent their formation.

## Material and methods

### Ethics

All study participants were thoroughly informed and provided explicit consent for the processing of their epidemiological data, in compliance with the requirements of the Clinical Research Ethics Committee at Fundación Jiménez Díaz University Hospital (FJD-SAC-16-01). All patients were informed about the study's objectives, the type of sample processing, and the anonymization of their personal data. After understanding all these points, they explicitly signed an informed consent form. In cases where the sialoliths were obtained from a minor, informed consent was obtained from the parent and/or legal guardian, who authorized both the processing and study of the sialoliths. All methods were carried out in accordance with relevant guidelines and regulations.

### Origin of the sialoliths

All sialoliths were collected by the Otolaryngology and Cervicofacial Pathology Department at the Hospital Universitario Fundación Jiménez Díaz, Hospital General Universitario de Villalba, and Hospital Universitario Infanta Elena. The stones were obtained through sialendoscopy, direct extraction, or spontaneous extrusion, depending on the case. For all cases, data was recorded regarding the involved salivary gland, the specific location of the lithiasis within the salivary system, and the method used to obtain the sialolith.

### Processing

Once collected, the stones were processed by the Geological Techniques Unit (Support Center for Research in Earth Sciences and Archaeometry) at the Complutense University of Madrid. The results pertaining to the mineralogical and chemical composition of the stones and their possible relationship with epidemiological factors were previously published by our research group (12).

The processing of the stones included an initial treatment with hydrogen peroxide for 48 hours to remove organic and/or blood residues from the surface, and to minimize potential bias due to bacterial contamination. The stones were then washed with Milli-Q water and air-dried.

Morphological studies were performed using a binocular magnifying glass (MOTIC SMZ-143-N2GG, 10X-40X) and a scanning electron microscope equipped with secondary electron, backscattered electron, and energy-dispersive x-ray detectors (SEM-EDX). The latter enabled chemical analyses. The samples that exhibited a complete, unfragmented conformation were selected for these analyses and they were mounted on a brass sample holder, specifically designed for the equipment used (JEOL JSM-820), using three different adhesives: graphite tape, glue, and silicone, always aiming to optimize the contact between the base of the sample and the surface of the holder. They were then dried in an oven at 45°C for 24 hours and coated with gold to ensure conductivity against the electron beam. For some exceptionally large sialoliths, polished specimens were prepared to observe cross-sections of the internal structure. These were embedded in epoxy resin and sectioned in half. The halves were polished to achieve a perfectly flat and mirror-like finish. For the remaining selected sialoliths, some fragments were separated for fresh-cut analysis, and both types of preparations were gold-coated.

The mineralogical composition of the stones was studied using x-ray diffraction (XRD) method. A Bruker D8 ADVANCE diffractometer was used with a Cu K*α* wavelength over an angular range of 2° to 65° 2*θ*, a step size of 0.02°, and a time per step of 1 s. Samples were dried at 45°C in an oven for 24 hours to remove any residual moisture. They were then ground in an agate mortar to obtain a fine powder (53 µm). The identification and semi-quantitative analysis of the phases present were performed using the XPowder program (13).

## Results

A total of 133 lithiasis specimens were collected from 102 patients, with an equal distribution between genders. In most cases, each patient presented with a single lithiasis (84.47%, *n* = 87), although up to 9 lithiasis were obtained from a single patient.

Most of the stones were from submandibular gland (78.2%, *n* = 104) and located in the ducts (80.45%, *n* = 107). Sialendoscopy, either alone or in combination with another approach, was the predominant method for obtaining the lithiasis (92.48%, *n* = 123). In one case, a lithiasis was obtained following a submandibular gland excision. The remaining stones were provided by the patients themselves after spontaneous extrusion (6.77%, *n* = 9). All data regarding the characteristics are summarized in Supplementary Table 1.

The mean age at the onset of obstructive symptoms was 45.83 years (range 6-88). When stratified into pediatric patients (<18 years) and adults (≥18 years), the mean age in adults was 47.72 years (range 20–88). Four lithiasis cases were of pediatric origin (4% of the sample), with a mean age of 10 years (range 6–14).

As previously published, the mean age of patients with parotid stones was significantly higher: 53.7 years for parotid stones, compared to 44.1 years for patients with submandibular stones (*p* = 0.031) (12).

### Macroscopic characteristics

**Figure 1:**
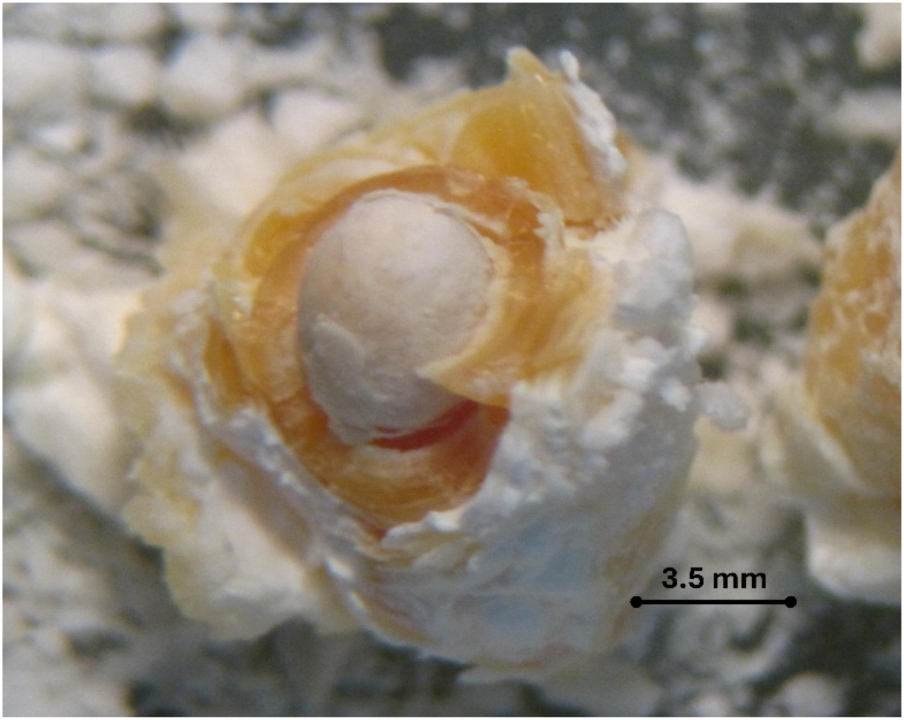
Image of a submandibular-ductal lithiasis obtained using a binocular magnifying glass. Magnification 25×. The macroscopic core, with its whitish coloration is surrounded by amber concentric layers.

All the studied sialoliths were oolitic aggregates, consisting of a superposition of concentric layers with different textures and colors, arranged around a well-defined core ( Figure 1 ). From a geological perspective, oolitic aggregates are spherical or subspherical forms that grow by the accretion of material around a core of any composition or morphology. They typically exhibit a concentric banded structure, though radial structures and combinations of radial and concentric structures are also known.

The morphologies of the sialoliths are variable: rounded, irregular, spheroidal, ellipsoidal, and globular (no polyhedral or faceted shapes were observed). Regarding their origin, we found that parotid stones are always irregular (almost elongated), regardless of whether they are ductal or hilar. In contrast, submandibular stones of ductal origin predominantly exhibit regular elongated shapes (ellipsoidal or cylindrical), with irregular spheroidal shapes being less common. No significant trends were observed in the hilar-origin submandibular stones.

Externally, the sialoliths exhibit a rough, botryoidal surface, with smooth surfaces found exceptionally. This surface may be cracked and/or encrusted. Additionally, they display different colorations, generally ivory-white and amber, distributed heterogeneously. Pure white color is rarely found.

The size and weight of these stones, determined in a previous study (12), have an average size of 5.44 mm and a median of 4.45 mm (size range 0.75–65.60 mm) and an average weight of 119.84 mg with a median of 48.4 mg (weight range 1.0–1,196.3 mg). The relationship between these parameters and the origin of the stones shows that the median size and weight values of submandibular stones are higher than those of parotid stones (5.00 mm vs. 3.75 mm and 58.8 mg vs. 21.1 mg).

### Mineralogical composition

The main crystalline phases identified by XRD in the 137 studied sialoliths were: octacalcium phosphate (OCP) Ca_8_H_2_(PO_4_)_6_·5H_2_O, hydroxyapatite (HAP) Ca_5_(PO_4_)_3_(OH), and whitlockite (WHL) Ca_3_(PO_4_)_2_ or Ca_9_Mg(PO_4_)·6PO_3_OH. Brushite (DCPD) CaHPO_4_·2H_2_O was detected in only a small proportion of the analyzed sialoliths.

All sialoliths exhibit extremely low crystallinity of the identified phases, particularly notable in the case of OCP. Additionally, they show a broad and intense background signal in the low-angle spectrum range (< 10°), attributed to the presence of amorphous phases and/or very low crystallinity. On average, the sialoliths studied contained 13.56% amorphous phase (Supplementary Table 2), possibly calcium phosphates (ACP), Ca_10__−x_H_2x_(PO_4_)_x_·(OH)_2x_, and organic matter. Only in one case, a ductal submandibular sialolith, was a flat background diffractogram obtained.

**Figure 2:**
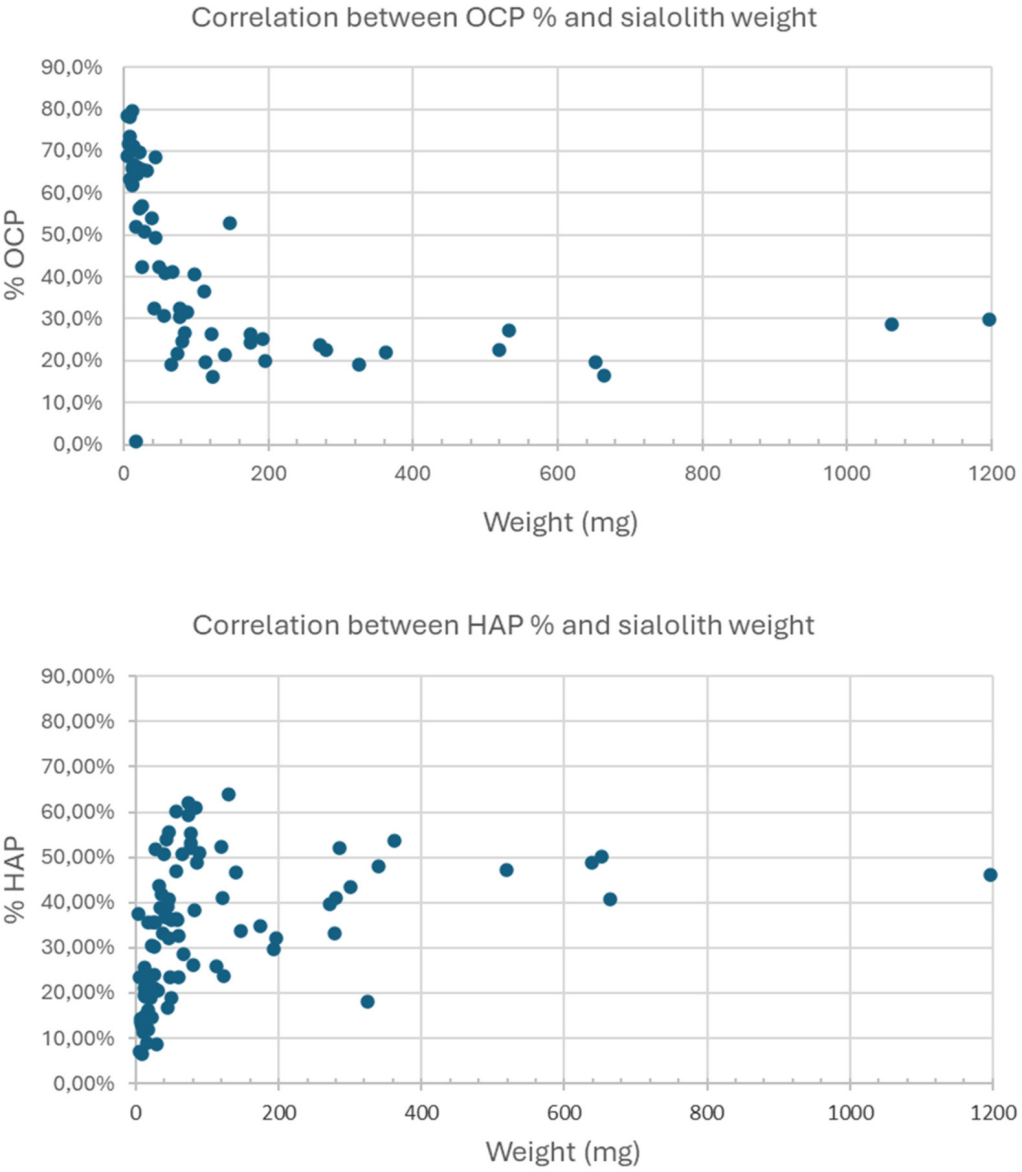
Relationship between the percentage of OCP (superior image) and HAP (inferior image) with the weight of the sialolith (mg).

Figure 2 shows the relationship between the proportion of OCP and HAP present in the sialoliths and their weight in mg. Two clearly opposite trends are highlighted: a decreasing trend in OCP and an increasing trend in HAP with the weight of the lithiasis.

### Microscopic characteristics: chemical composition and internal structure

This section presents the morphological analysis along with the results of the 483-point chemical analyses performed using SEM-EDX on 48 sialoliths.

**Figure 3:**
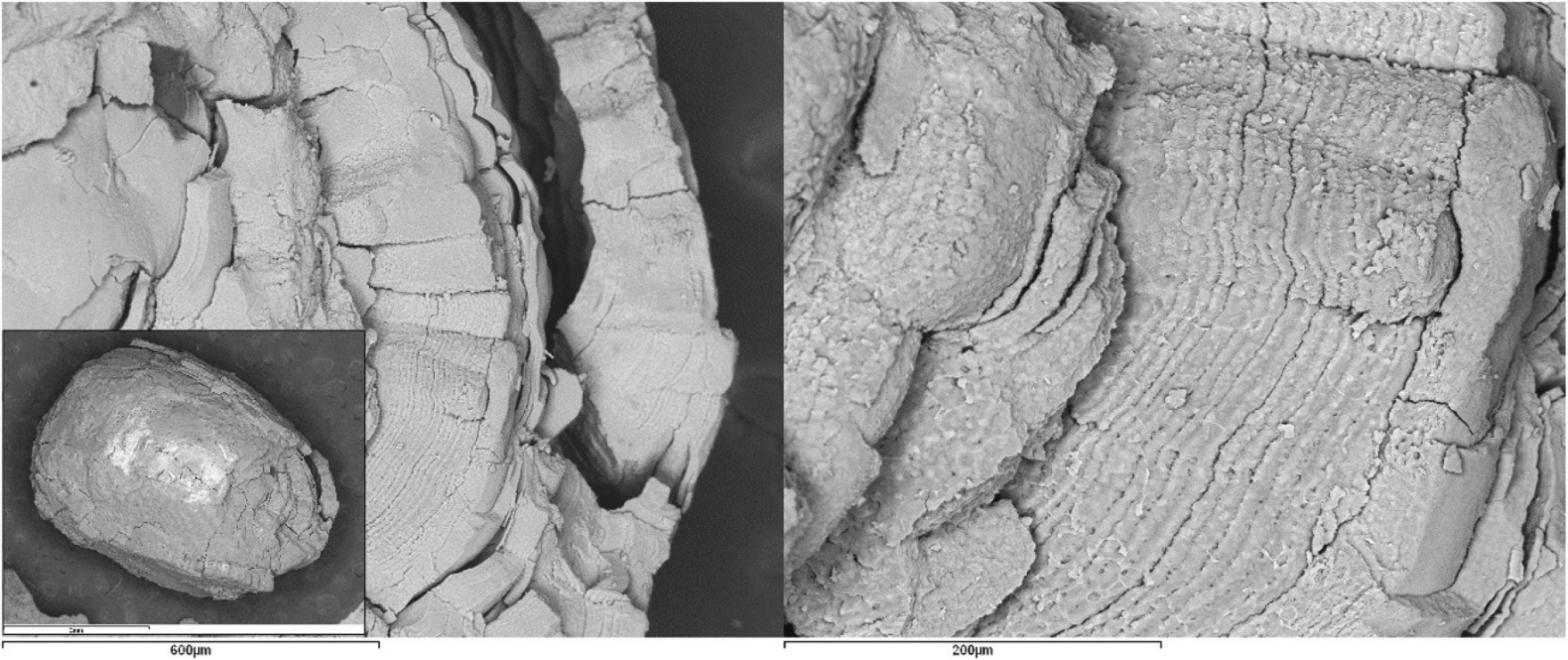
SEM images with BSE of a ductal submandibular lithiasis. Left image: Core (Magnification 25×) and external concentric macrolayers with scalloped contacts (Magnification 100×). Right image: Detail of the macrolayers (Magnification 300×).

**Figure 4:**
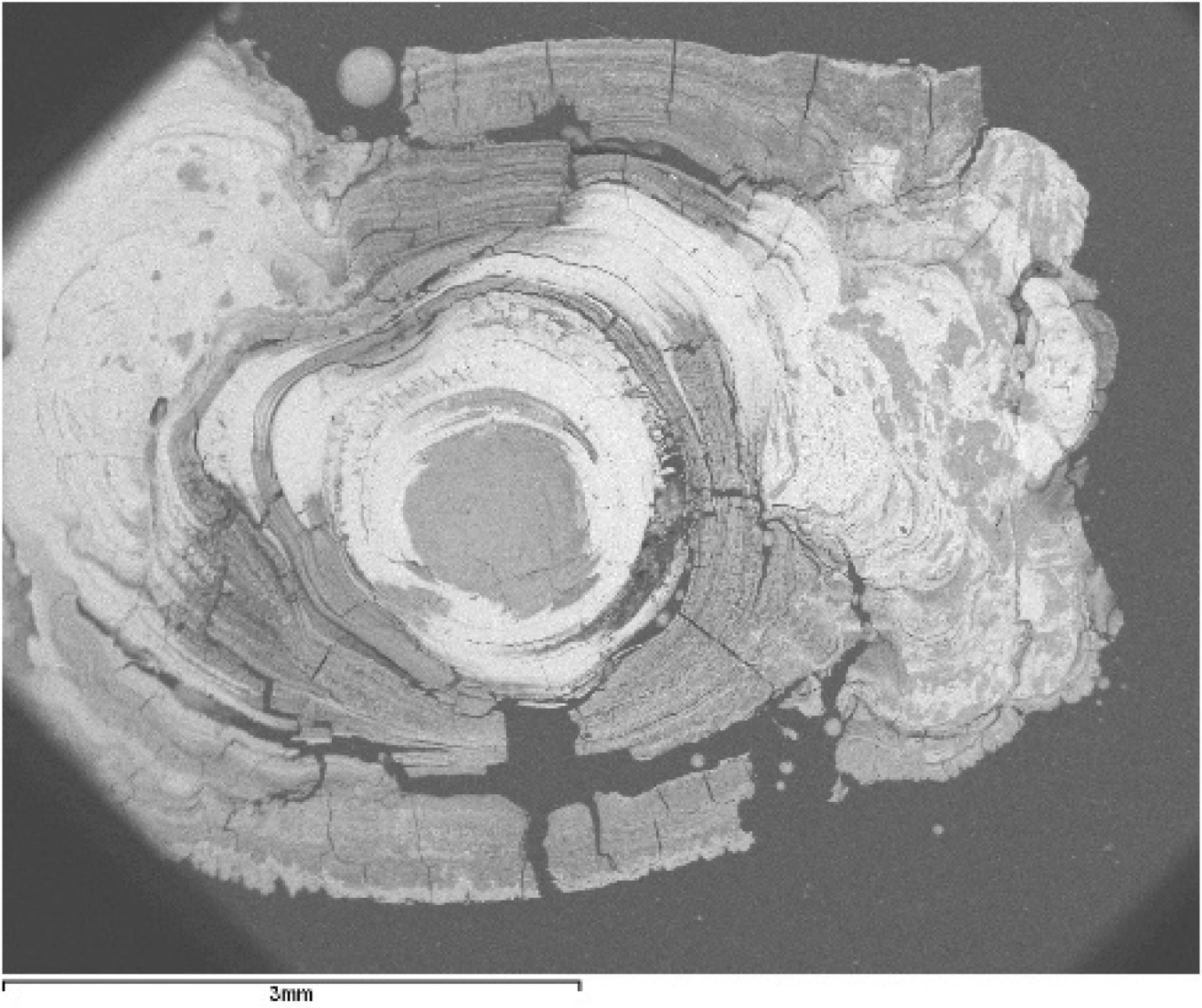
SEM image with BSE of a ductal submandibular lithiasis (magnification 20×). Oolitic texture. Core and concentric light and dark layers.

In all cases, it was confirmed that the internal core, like its external shell, has a concentric structure formed by very thin and compact layers that increase in thickness and acquire a coarser texture towards the exterior. Generally, as the volume of the aggregate increases, it becomes more heterogeneous, interspersing levels or lenticular plates of dark color with other light-colored layers (observed through BSE) and various morphologies, with variable thicknesses and different textures. These layers are grouped into macrolayers, among which numerous cracks and fractures are observed ( Figures 3, 4 ).

### Chemical composition

The gold metallization of the sialoliths for SEM-EDX analysis has allowed for the identification and determination of the carbon present in them. EDX analysis of the darker-colored levels and formations has identified carbon (C) as the major element in 100% of the analyzed specimens, and sulfur (S) in only 40% (in a lower proportion than C and always associated with it). The presence of both elements is associated with organic matter. Supplementary Table 3 shows the relationship between the origin of the sialoliths and their content of these two elements.

It has also been determined that C constitutes an average of 30% of the weight in submandibular sialoliths (ductal and hilar), whereas no significant trend could be established for parotid sialoliths (Supplementary Figure 1).

The chemical analysis of the lighter-colored areas has identified calcium (Ca) and phosphorus (P) in proportions compatible with the stoichiometry of some mineral phases identified by XRD. These elements appear together in most of the studied stones. Magnesium (Mg) was also detected in 39.6% of the analyzed sialoliths. This element may be part of the composition of WHL, which was identified in 79% of the stones containing Mg. In the remaining 21%, Mg-containing WHL could also be present, although in a proportion below the x-ray detection limit (< 5%).

Supplementary Figure 2 shows the average Ca/P ratio from the chemical analyses performed. It is observed that this ratio aligns preferentially with those of HAP (1.67) along with calcium-deficient hydroxyapatite (CDHA) [Ca_10__−x_(HPO_4_)_x_(PO_4_)_6__−x_(OH)_2__−x_] and WHL (1.5), followed by octacalcium phosphate (OCP) (1.33).

**Figure 5:**
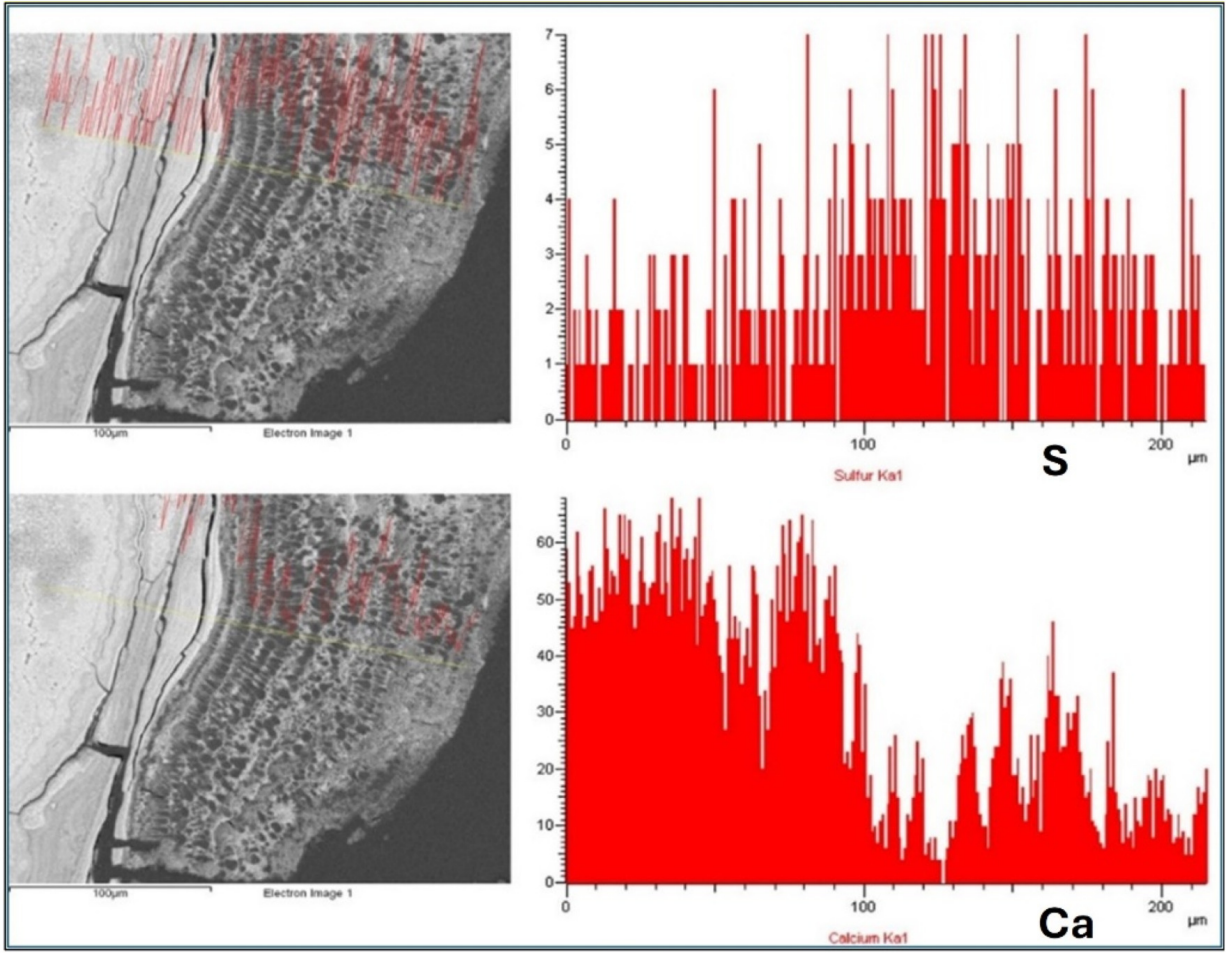
Composition profiles corresponding to S and Ca, determined by EDX (intensity in arbitrary units) in the cortex of a sialolith. The dark-colored layers contain a higher concentration of S (upper profile) than the light-colored layers, in which Ca predominates (lower profile).

To confirm the relationship between the organic/inorganic content and the grayscale tones (dark/light) of the materials, composition profiles of P, Ca, C, and S were performed using EDX on the cortex of a sialolith that clearly shows this color difference. The obtained profiles indicate a high content of C and S in the dark layers and a higher concentration of Ca and P in the light layers ( Figure 5 ).

### Internal structure

**Figure 6:**
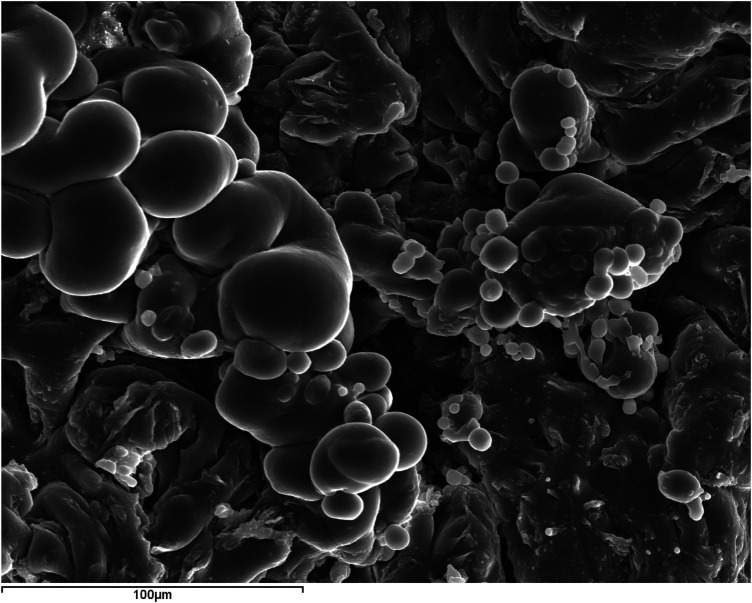
SEM image with SE of submandibular ductal lithiasis (magnification 500×). Organic spherules in a fragment of a dark concentric layer composed of 100% C.

**Figure 7:**
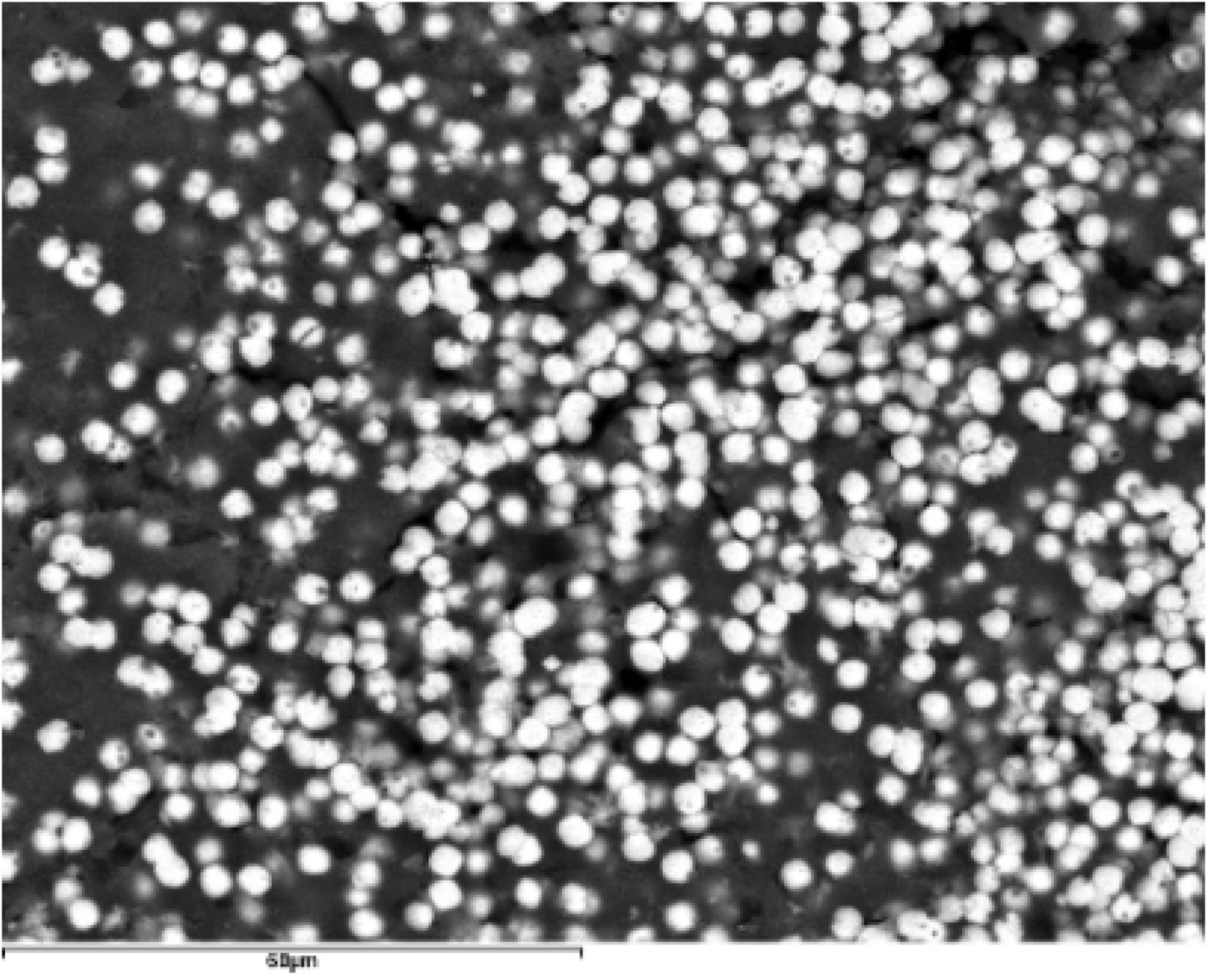
SEM image with BSE of submandibular hilar lithiasis (magnification 1000×). Sialomicroliths (white spheres: Ca/P = 1.37) immersed in a dark organic matrix with a high S content.

The organic matter (dark in color), although it can appear massive and homogeneous, more frequently exhibits pronounced spheroidal shapes ( Figure 6 ). Around these organic spherules, other inorganic phases (light in color) are arranged, eventually covering them completely. The study of polished specimens has allowed the observation of the internal core of some sialoliths, consisting of aggregations of sialomicroliths (white) immersed in a dark organic matrix ( Figure 7 ).

**Figure 8:**
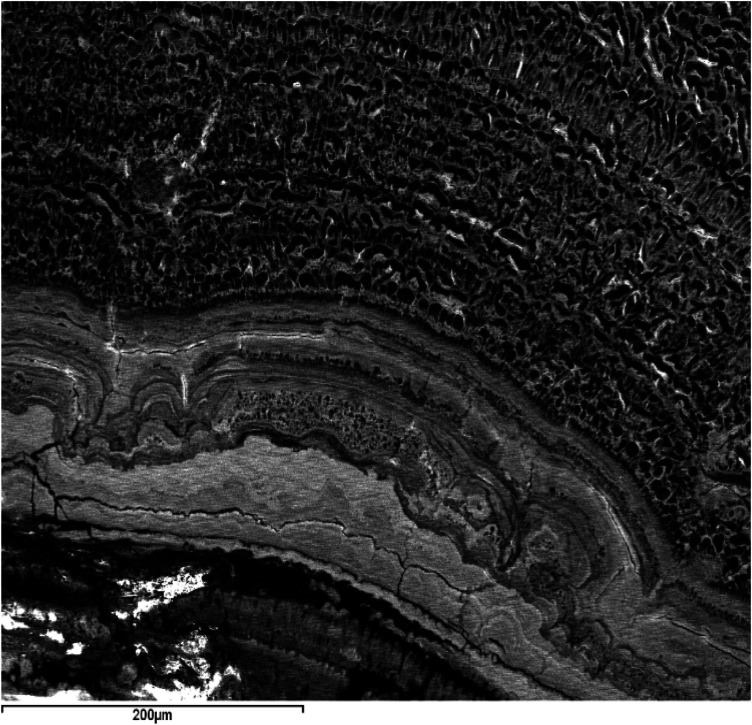
SEM image with BSE of a submandibular hilar lithiasis (magnification 250×). Dark layers formed by deformed organic matter spherules encapsulated by calcium phosphates. Light layers in which dark spherules and small oolites can be distinguished, cemented by a massive-looking matrix with scalloped contacts.

**Figure 9:**
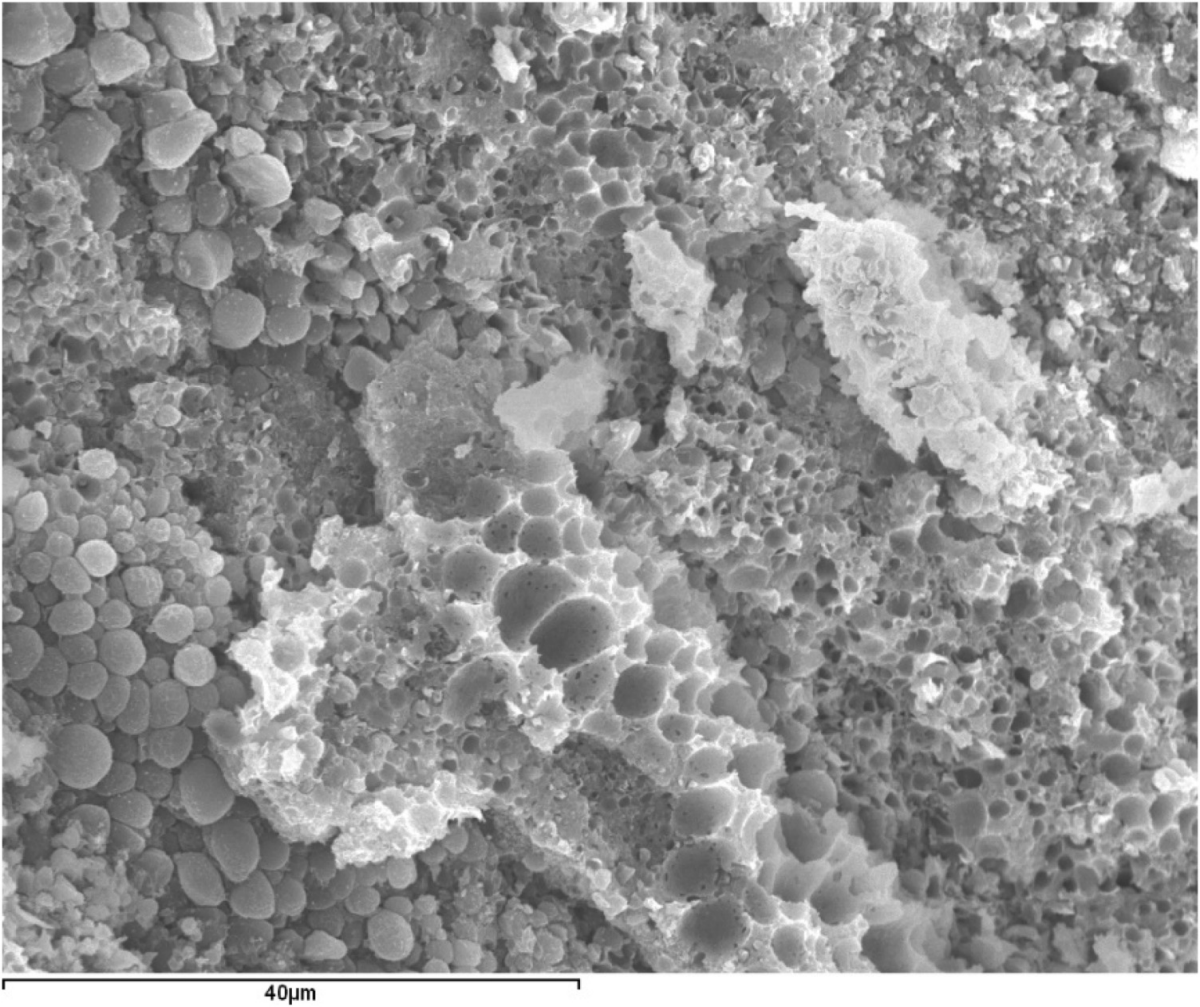
SEM image with SE of a submandibular hilar lithiasis (magnification 1500×). Empty molds of calcium phosphates that may have been occupied by organic spherules.

Layers of sialoliths formed by organic spherules encapsulated in a thin coating of calcium phosphate can be observed. These spherules may be deformed and adopt elongated shapes ( Figure 8 ). Hollow alveoli are often seen, which could correspond to empty molds of the spherules, formed by the calcium phosphate that encapsulates them ( Figure 9 ).

**Figure 10:**
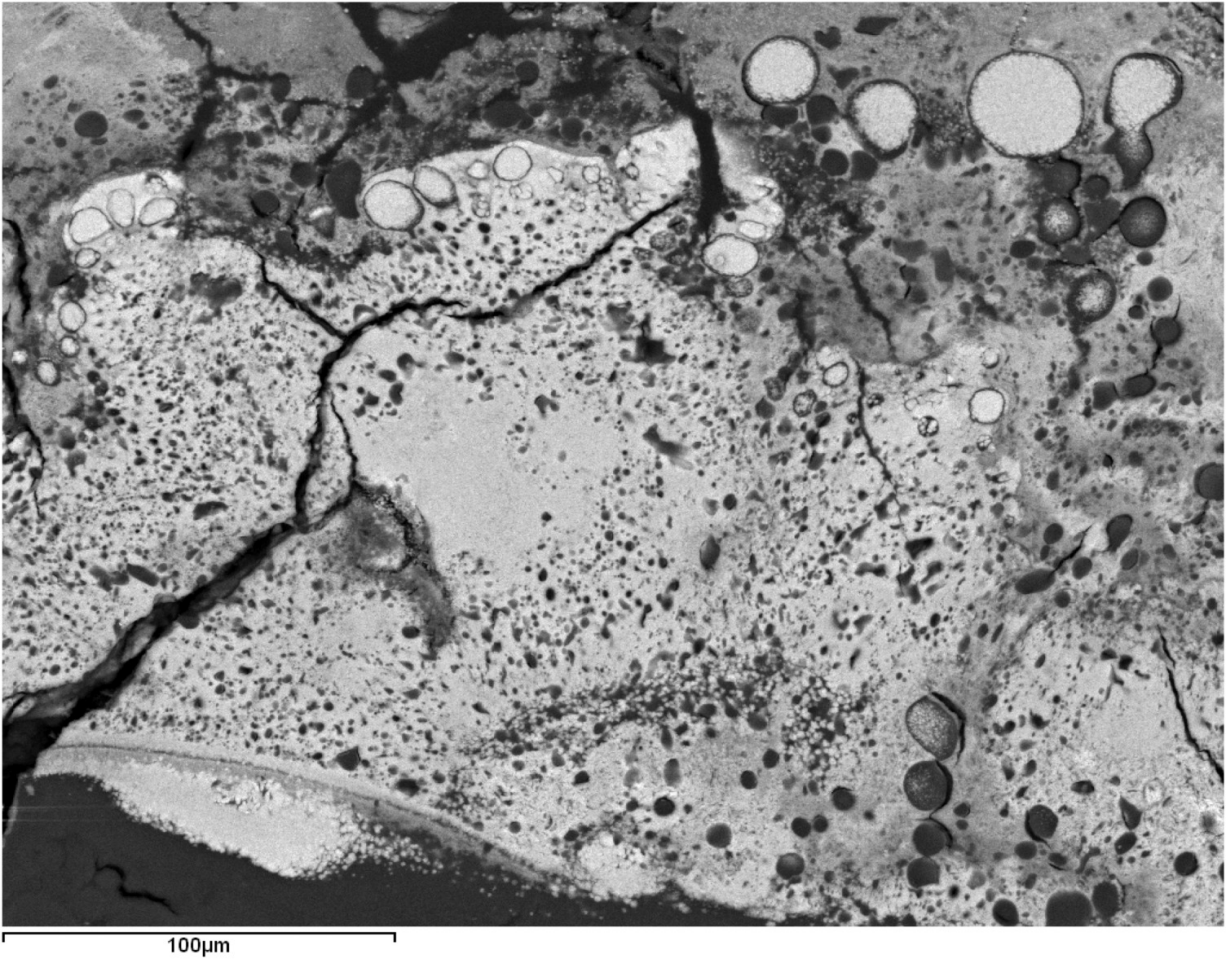
SEM image with BSE of a submandibular hilar lithiasis (magnification 400×). Macroscopic core-concentric layer contact. Organic spherules (dark circles) and sialomicroliths (white circles) cemented by a very light-colored massive-looking calcium phosphate matrix. The gray spherules correspond to intermediate degrees of calcification, with high contents of C and S (organic) in addition to Ca and P (inorganic). In the white sialomicroliths, a Ca/P ratio of ≈1.45 has been determined (slightly lower than that corresponding to WHL and CDHA ≈ 1.5).

**Figure 11:**
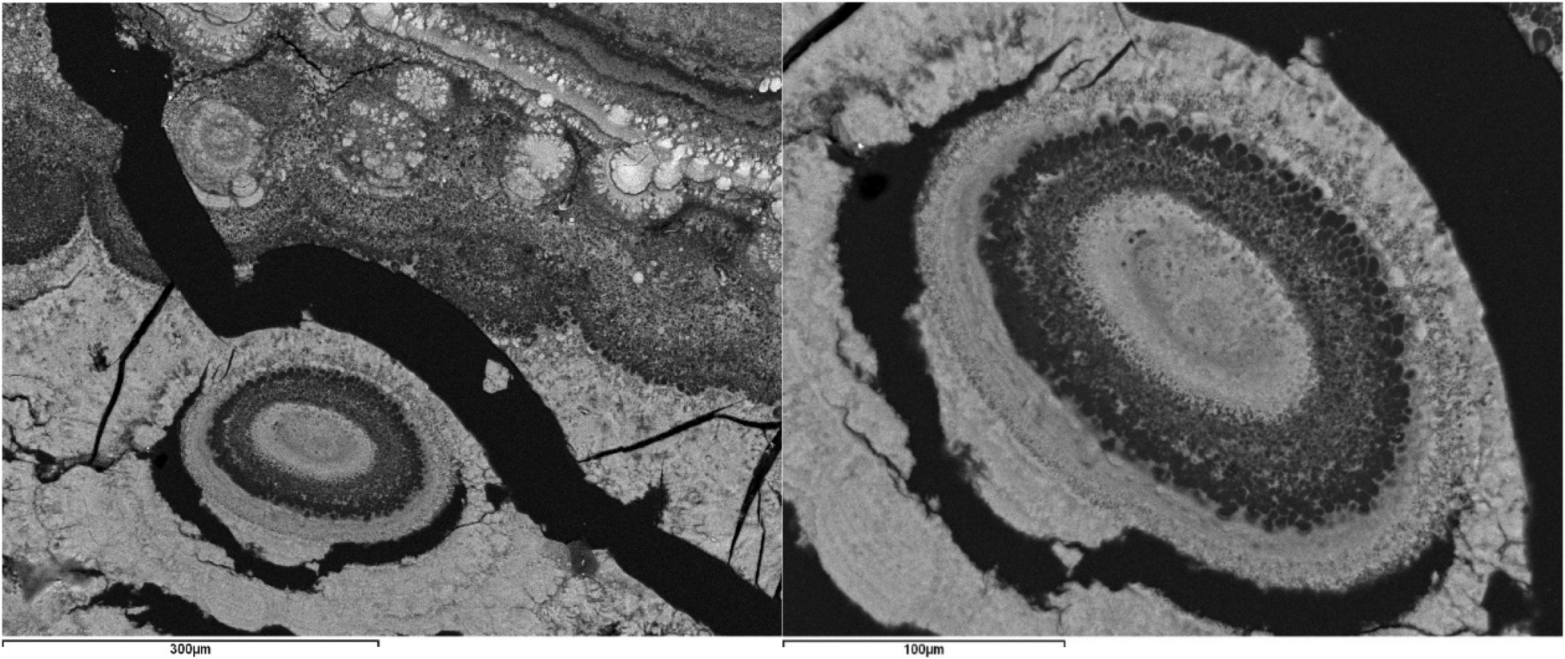
SEM image with BSE of a submandibular ductal lithiasis. Left image: Light and dark layers containing smaller oolitic aggregates and sialomicroliths (Magnification 200×). Right image: Detail of an oolite formed by concentric light layers composed of sialomicroliths and dark layers composed of spherules with surface calcifications (Magnification 450×).

Calcium phosphates also form the massive cement that binds various formations such as organic spherules (dark) and sialomicroliths with different degrees of calcification, detectable by their varied tones, ranging from dark grey (less calcified) to white (more calcified) (Figure 10). Additionally, it can bind other oolitic aggregates, responsible for the scalloped contacts often observed between the different layers (Figures 8, 11).

**Figure 12:**
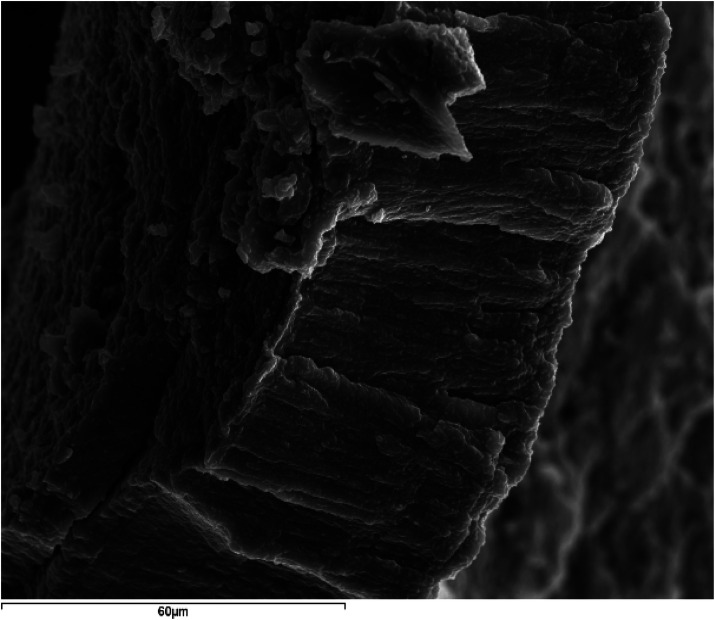
SEM image with SE of a submandibular ductal lithiasis (magnification 1000X). Palisade texture.

**Figure 13:**
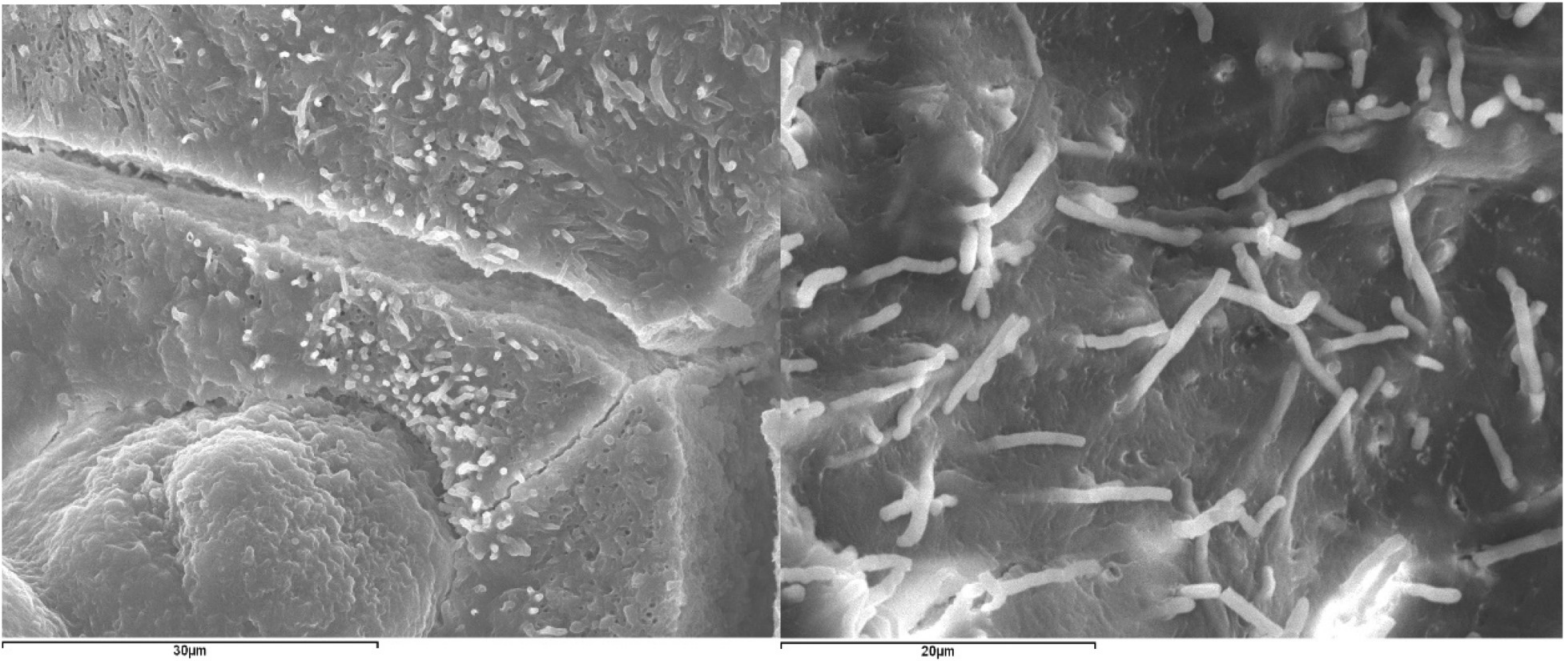
Bacillary morphologies in sialoliths. Left: SEM image with SE of a submandibular ductal lithiasis (Magnification 2000×). Right: SEM image with SE of a parotid-ductal lithiasis (Magnification 2500×).

**Figure 14:**
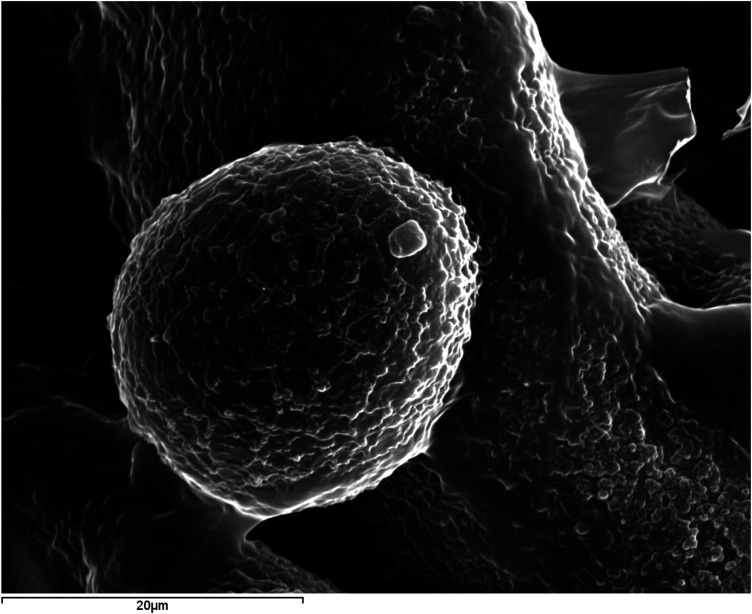
SEM image with SE of a parotid-ductal lithiasis (magnification 2500×) showing an organic spherule (C = 71.6–89.7%) with bacterial morphologies observed on its surface.

Finally, faceted crystals were occasionally observed, palisade textures (Figure 12), and bacillary morphologies as shown in Figure 13. Both correspond to two sialoliths of submandibular ductal and parotid ductal origin, respectively. Figure 14 shows an organic spherule with a series of grouped granules forming a kind of high-relief reticulum resembling bacterial morphologies.

## Discussion

Although the studied sialoliths show significant differences, which is expected given their diverse origins and formation conditions, their morphological and compositional characteristics exhibit common patterns.

Firstly, all are oolitic crystalline aggregates, characterized by the presence of a core around which a succession of concentric layers is arranged, combining organic and inorganic compounds (Figure 1). Sodnom-Ish et al. (4) describes a similar conformation in the sialoliths they studied, although they distinguish three well-defined zones: a central core, an intermediate compact band, and a peripheral multilayered zone. This intermediate band has been identified in very few specimens in our study. The limited number of complete cross-sections we have been able to observe in our sialoliths does not allow us to categorically confirm or refute this observation. However, there is a clear differentiation between the core and the outer shell, which we have attributed to the loss of cohesion in the sialolith, a consequence of the increasing thickness of the layers as they move away from the center (formation of macrolayers), their heterogeneous nature (combining different textures and materials, both organic and inorganic), and the fractures that originate between them (Figures 3, 4).

These authors also consider that the initiation of the sialolith occurs in the core, stemming from a bacterial infection that produces a biofilm, inducing calcification and forming the compact band they describe in the intermediate zone. This hypothesis could not be confirmed in our study, as we detected the presence of the intermediate band only occasionally. Therefore, it is important to note that bacterial theory still remains controversy regarding whether these microorganisms are primary causative agents or secondary consequences of obstructive and inflammatory processes. Harrison strongly argues that bacterial presence in sialoliths is secondary to obstruction and inflammation rather than their primary cause, based on extensive experimental and clinicopathological studies demonstrating that inflammation and obstruction precede significant bacterial colonization in most cases (14).

However, we found evidence of a similar process around spherules, also referred to as vesicles or exosomes (4, 15). These spherules are observed in practically all fragments of organic matter studied via SEM, whether originating from the core or any other region of the sialolith (Figure 6). They are frequently seen encapsulated by calcium phosphates, partially or completely, forming sialomicroliths in the latter case (Figures 7–10).

Figure 14 shows what could be equivalent to the biofilm described by Sodnom-Ish et al. (4), but located around an independent spherule, which could act as a scaffold for the nucleation of calcium phosphate, eventually forming the sialomicrolith at the final stage of its development. In detail, from the perspective of crystal growth, the crystallization of any substance requires a specific concentration of constituent elements (Ca, P, Mg, etc., in this case) known as supersaturation, and the prior formation of a nucleus or cluster of the atoms that make up the substance, which must reach a critical size to grow. This process is called nucleation, and when it occurs on a preexisting scaffold, as suggested for the formation of sialomicroliths, it is referred to as heterogeneous nucleation (16).

Kraaij et al. (1) describes substructures within the macroscopic core, formed by the association of smaller structures and the superposition of different processes, such as agglomerations of sialomicroliths and nucleation on mucoid materials. Figure 7 corresponds to the interior of the macroscopic core of a sialolith from our study, which is as complex as that described by Kraaij et al. (1). It shows agglomerations of sialomicroliths immersed in an organic matrix, probably mucoid material. Detailed structural investigations by computed microtomography have revealed that the primary nucleus of sialoliths does not exhibit a single mineralization pattern; instead, it can be predominantly mineralized or largely organic. The subsequent growth of the calculus occurs through concentric layering or irregular patterns, reflecting complex physicochemical processes such as surface tension dynamics and Liesegang-Ostwald phenomena (17).

Im et al. (18) describe zones with different colorations in sialoliths, related to their organic or inorganic content, similar findings were observed in our study. Among the organic components, they identified collagen, glycoproteins, free amino acids, carbohydrates, and a high sulfur content, which they associate with the secretory activity of the salivary glands. Indeed, it is plausible that the high presence of sulfur can be justified by the degradation of organic components over time, such as proteins. Several studies indicate that sialomicroliths, although commonly present in healthy salivary glands, do not directly serve as immediate precursors to larger salivary stones (sialoliths). Rather, these small structures may cause focal obstructions, leading to local glandular atrophy. This atrophy facilitates the accumulation of organic and inorganic materials, as well as secondary bacterial infections, creating an inflammatory vicious cycle that eventually culminates in the formation of larger salivary stones (19, 20).

On the other hand, Im et al. (18) also study the texture of the concentric layers and find that those with a more inorganic composition consist of globular aggregates of sialomicroliths with different degrees of calcification. They identify an amorphous interior composed of organic material in direct contact with a superficial inorganic layer formed by magnesium whitlockite and hydroxyapatite. Figure 10 shows a detail of the contact between the macroscopic core and the first layer of the shell in a sialolith. In it, a white inorganic cement (calcium phosphate) is observed, encompassing numerous dark spherules, purely organic, and sialomicroliths with varying degrees of calcification, detectable by their varied tones, from dark grey (less calcified) to white (more calcified). In other specimens from our study, palisade textures (Figure 12) have been observed, which could result from the calcification of deformed and elongated organic spherules (Figure 8). Smaller sialoliths have also been observed embedded within the layers (Figures 8, 11). All these rounded forms cause the layers to have undulated surfaces on which other layers overlap, which must also undulate to adapt to them, resulting in the scalloped contacts often observed between layers (Figure 8). The botryoidal surface characteristic of sialoliths would be the external manifestation of these undulated surfaces, scalloped contacts, and even smaller sialoliths that may be coupled in their outermost layer. Conversely, smooth surfaces are exceptional.

Kraaij et al. (1) consider that the shape and surface roughness of sialoliths depend on their anatomical or glandular origin. Those of ductal origin are mostly elongated, while those from the hilum or parotids are round or oval. Submandibular stones generally have a smoother surface, while those from the parotids are more irregular. However, the specimens studied in this work exhibit such variability that it has not been possible to establish clear relationships with their origin, except for very general trends: parotid stones tend to be irregular, and ductal submandibular stones tend to be more regular. Regarding surface characteristics, the heterogeneity is such (apart from the predominantly botryoidal habit) that any attempt to classify or group the population of sialoliths studied in this work is impossible.

Regarding the crystallinity of these calcifications, the presence of amorphous phases has been confirmed in all cases. These amorphous phases are related in the literature to the organic content (18) or to certain amorphous or very low-crystallinity inorganic compounds (21). These compounds initially precipitate and evolve over time into more crystalline phases through aging or Ostwald Ripening phenomena (16, 22). This phenomenon involves the dissolution of smaller crystals or amorphous phases and the redeposition of the dissolved species on the surfaces of larger crystals. As the process continues, smaller particles reduce and eventually disappear, while larger ones grow, generally increasing the average size and crystallinity. Eventually, the entire population of particles could transform into a single large crystal.

In our work, the results obtained from the comparative study between the weight of the sialoliths and the percentage of OCP and HAP effectively support the aging hypothesis (23, 24), as they show a clear decrease in the less crystalline phase (OCP) and a clear increase in the more crystalline phase (HAP) as the weight of the sialolith increases (Figure 2), and therefore, the evolution time.

On the other hand, the fact that only one flat diffractogram was obtained in the entire population of sialoliths studied, and that its chemical analysis revealed a high sulfur content, indicates the presence of organic matter as a principal constituent. This would support the hypothesis of Kraaij et al. (1), who consider that sialoliths formed exclusively of organic matter, or with a high content of it, are extremely rare. They assert that the most common composition is predominantly inorganic materials, with the proportion varying according to the origin of the stone. In our study, it was found that organic matter, primarily associated with the presence of carbon, constitutes an average of 30% in submandibular stones, without being able to establish any significant trend in those of parotid origin (Supplementary Figure 1).

The great complexity and morphological and compositional variability observed in the studied sialoliths could result from formation mechanisms comparable to those described by Sivaguru et al. (11) for renal stones. These authors suggest episodes of nucleation on different types of substrates and the growth, dissolution, recrystallization, and aggregation of various crystallizations to form a single calcification. This process could even occur in several stages and intermittently, similarly to the natural processes that have governed biomineralization on Earth for billions of years. Consistent with this complexity, previous studies emphasize that inflammation plays a crucial role in the progression of sialadenitis and in the secondary formation of larger sialoliths. Chronic inflammation, triggered by recurrent micro-obstructions caused by sialomicroliths, leads to progressive histological changes such as glandular atrophy and fibrosis, thus favoring the secondary formation of larger stones (20, 25).

Regarding the epidemiological and morphological characteristics, it is noteworthy that parotid lithiasis tends to occur in older patients compared to submandibular lithiasis. As previously stated (12), possible explanations for the higher age observed in patients with parotid stones include anatomical factors, metabolic differences, or deliberate surgical delay due to patients’ concerns about potential complications of the procedure. In addition, the median size and weight values of submandibular stones are higher than those of parotid stones. This difference may reflect anatomical and gravitational factors, including the presence of longer and more tortuous ducts that favor progressive mineral accretion.

While our study provides relevant insights into sialolith formation mechanisms, comprehensive preventive strategies remain partially addressed, warranting further research. One of the main limitations of our study is the relatively small sample size (133 lithiasis from 102 patients), which may not fully represent the broader population of individuals affected by sialolithiasis. The specific nature of our sample population, derived from hospital patients, may introduce selection bias and limit the generalizability of our findings. Another important limitation is related to the size of the studied sialoliths, which generally do not reach the minimum size necessary to perform complete cross-sections to observe the different elements that comprise them and to conduct the relevant statistics. By other hand, there are some limitations inherent to the analytical techniques, such as in the case of XRD, which, especially in samples with low crystallinity, has a detection limit of 5%. Finally, the variability in crystallinity and the influence of external factors such as diet, hydration status, and genetic predisposition on sialolith formation were not fully explored, warranting additional research to address these aspects comprehensively.

### Proposed model

**Figure 15:**
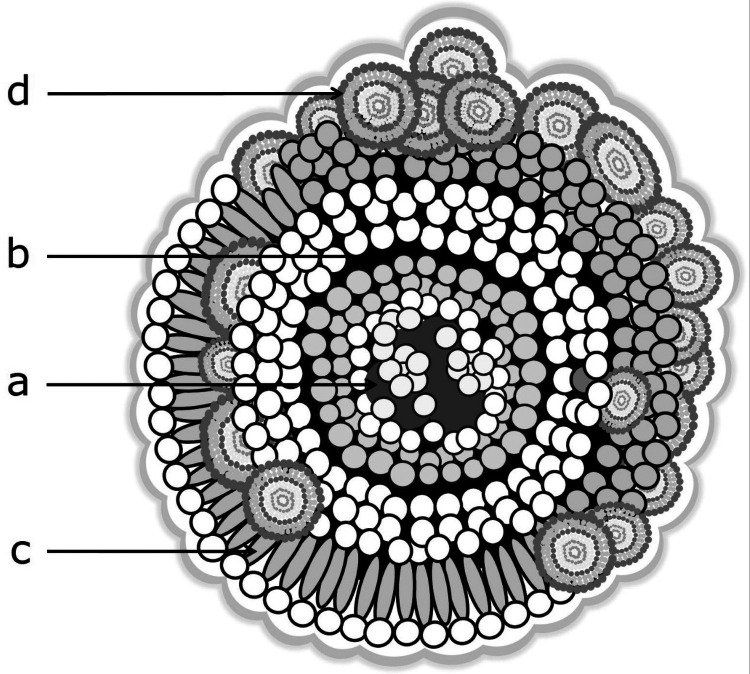
Ideal structure of a sialolith. The different shades of grey indicate the differences between organic spherules (dark) and sialomicroliths (white). (a) Core: sialomicroliths and exosomes immersed in an organic material. Encased in a succession of thin, compact concentric layers. (b) The aggregate grows and loses homogeneity and cohesion. The layers become thicker, and the core separates from the outer shell. (c) Organic spherules arrange in columns or deform, elongating perpendicularly to the layer, resulting in palisade texture upon calcification. (d) The sialoliths that form the outermost layer are responsible for the botryoidal morphology of the calculus surface.

Based on the results obtained and the existing literature to date, a novel conformational model for salivary origin sialoliths can be developed (Figure 15):

1. Formation of Initial Calcifications: Heterogeneous nucleation of calcium phosphates occurs on organic spherules (exosomes) within an organic matrix (rich in C and S). These spherules agglomerate and form the central core of the aggregate. Nucleation is favored by the presence of a biofilm around the spherules.
2. Layer Accretion Around the Proto-Core: Layers can form around the proto-core in various ways: a) Formed by organic spherules, calcified. b) Formed by sialomicroliths. c) Formed by a continuous precipitate of calcium phosphate around the central core. d) Formed by a continuous precipitate of calcium phosphate around already formed layers.
3. Core Volume Increase: The central core increases in volume by repetition of step 2.
4. Inclusion of Different Elements: Simultaneously with step 3, different elements can be included, such as other developed sialoliths, layers of organic matter with spheroidal and/or elongated morphologies. Upon calcification, these elements are responsible for the palisade textures occasionally seen in some layers.
5. Formation of Thicker Macro Layers: The different layers associate into thicker macro layers.
6. Increased Heterogeneity: Repetition of steps 3 and 4 increases the overall heterogeneity. This situation leads to the loss of cohesion of the aggregate and the separation of the core from the outer layers.
7. Development of a Rough-Botryoidal Surface: This surface reflects the spheroidal aggregates that form the outermost layer, that is, sialoliths of different volumes.
8. Substrate for New Layer Accretion: The surface developed in step 7 can serve as a substrate for the accretion of new layers, leading to an increase in the volume of the aggregate.
9. Improvement of Crystallinity: Simultaneously with the previous steps, the crystallinity improves through an Ostwald-Ripening process.

## Conclusions

The studied sialoliths are oolitic crystalline aggregates, characterized by a well-differentiated core around which a succession of concentric layers of organic and inorganic compounds is arranged.

Calcification begins as a process of heterogeneous nucleation around organic spherules (vesicles or exosomes) induced by the presence of a biofilm, which is generated around the spherule and may result from interactions with bacterial germs. The rather calcified spherules aggregate to form a proto core, upon which layers of new spherules, other sialoliths, and continuous calcium phosphate precipitates are added. As the layers move away from the core center, they become more complex and thicker. The sialolith loses cohesion, and the core becomes well differentiated from the rest. Over time, the lithiasis increases in volume while undergoing an Ostwald-Ripening process, which increases its crystallinity. The result is an oolitic aggregate with a rough, botryoidal surface and a degree of crystallinity that depends on the size of the aggregate.

A novel conformational model developed by our research group determines that lithiasis depends on the relationship between organic matter, inorganic matter, bacterial germs in the form of biofilms, and time. Despite the variable origin of the lithiasis and the obvious interpersonal differences, all analyzed stones conform to this multilayer conformational model, influenced by time in the crystallinity of the inorganic phases involved in the process.

## Data Availability

The original contributions presented in the study are included in the article/Supplementary Material, further inquiries can be directed to the corresponding author.

## Supplementary material

Supplementary Figure 1 Relationship between C content (% weight) and the origin of the sialoliths: **submandibular** ductal (SD), **submandibular** hilar (SH), and parotid ductal (PD). Total sample: 48 sialoliths. Number of analyses: 245.

Supplementary Figure 2 Ca/P ratio determined by EDX for all analyses performed. The highest concentration of points corresponds to the range between 1.5 and 1.75, which includes WHL and CDHA (1.5) and HAP (1.67); followed by the region between 1.3 and 1.5, which includes OCP (1.33).
