# Supplementary material for: Evolutionary conformation model of salivary gland lithiasis
Source: Front Oral Health. 2025 Jun 5;6:1610977. doi: 10.3389/froh.2025.1610977 (PMC12176897; doi:10.3389/froh.2025.1610977)
Supplement: Supplementary file 1 [file Table1.docx]

| Characteristic | % (n) |
| --- | --- |
| Patient gender |  |
| Male | 51,96% (53) |
| Female | 48,08% (49) |
| Number of lithiasis per patient |  |
| 1 lithiasis | 84,47% (87) |
| 2 lithiasis | 10,68% (11) |
| 3 lithiasis | 1,94% (2) |
| 4 lithiasis | 0,97% (1) |
| 5 lithiasis | 0,97% (1) |
| 9 lithiasis | 0,97% (1) |
| Affected gland |  |
| Submandibular gland | 78,2% (104) |
| Parotid gland | 21,8% (29) |
| Location in salivary system |  |
| *Ductal* | 80,45% (107) |
| *Hilar* | 17,29% (23) |
| *Intraglandular* | 2,26% (3) |

Table 1: Characteristics of the obtained lithiasis
