# Supplementary material for: Evolutionary conformation model of salivary gland lithiasis
Source: Front Oral Health. 2025 Jun 5;6:1610977. doi: 10.3389/froh.2025.1610977 (PMC12176897; doi:10.3389/froh.2025.1610977)
Supplement: Supplementary file 3 [file Table3.docx]

|  | OCP | HAP | WHL | DCPD | ACP |
| --- | --- | --- | --- | --- | --- |
| % sialoliths per mineralogical phase | 90,6 | 91,0 | 55,0 | 2,9 | 100 |
| % Weight (average) | 41,5 | 32,98 | 11,25 | 0,71 | 13,56 |

Table 2: Distribution of phases identified by XRD in the total sample of sialoliths and the averaged weight percentage for each phase.
