# Supplementary material for: Evolutionary conformation model of salivary gland lithiasis
Source: Front Oral Health. 2025 Jun 5;6:1610977. doi: 10.3389/froh.2025.1610977 (PMC12176897; doi:10.3389/froh.2025.1610977)
Supplement: Supplementary file 4 [file Table4.docx]

| Element | % SD | % SH | % PD |
| --- | --- | --- | --- |
| C | 61,6 | 7,0 | 15,4 |
| S + C | 8,4 | 6,0 | 1,6 |

Table 3: Relationship of the presence of carbon (C) and sulfur (S) with the origin of the sialoliths in a sample of 48 sialoliths. Submaxillary Ductal (SD), Submaxillary Hilar (SH), and Parotid Ductal (PD)
