# Supplementary figures and images for: Evolutionary conformation model of salivary gland lithiasis
Source: Front Oral Health. 2025 Jun 5;6:1610977. doi: 10.3389/froh.2025.1610977 (PMC12176897; doi:10.3389/froh.2025.1610977)

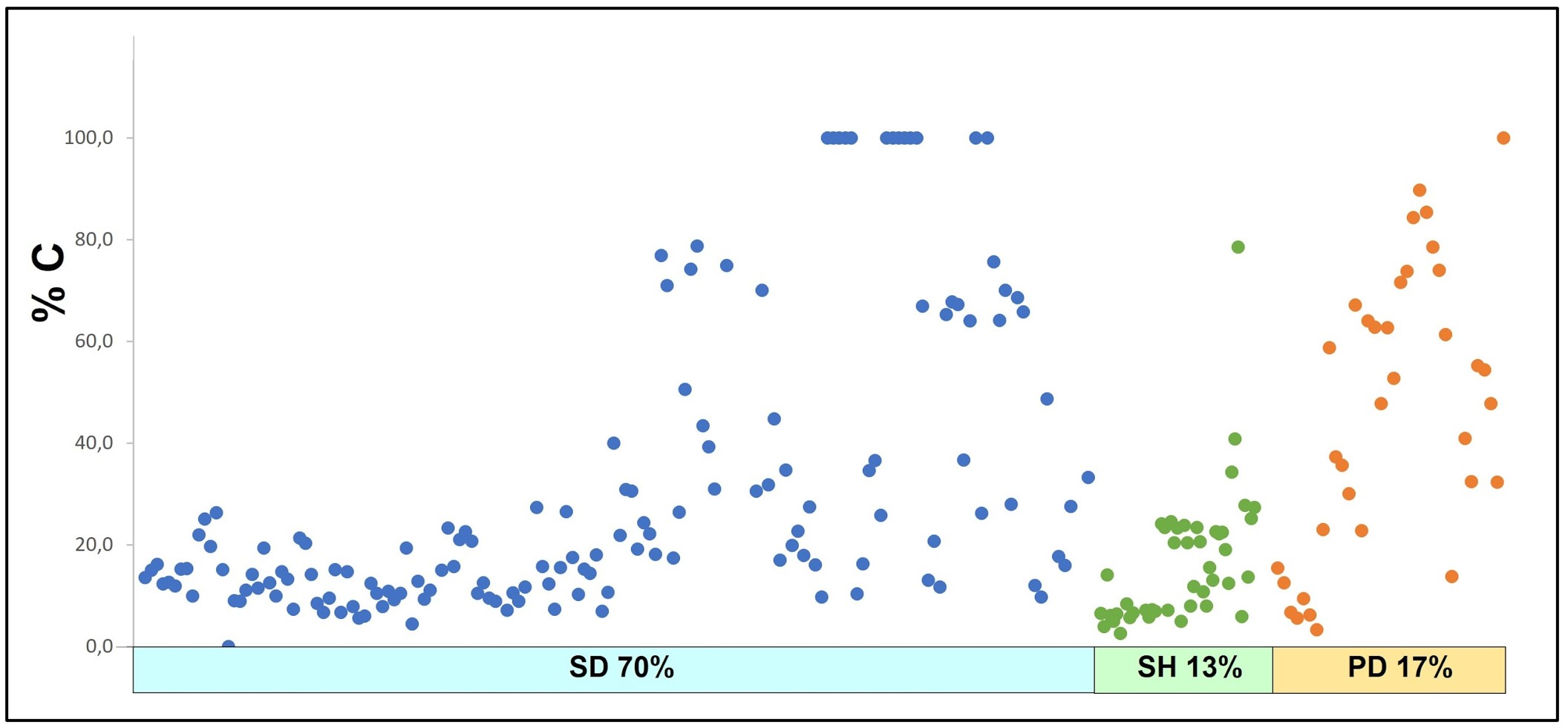

Supplement: Supplementary Figure 1 — Relationship between C content (% weight) and the origin of the sialoliths: submandibular ductal (SD), submandibular hilar (SH), and parotid ductal (PD). Total sample: 48 sialoliths. Number of analyses: 245. [file Image1.tiff]

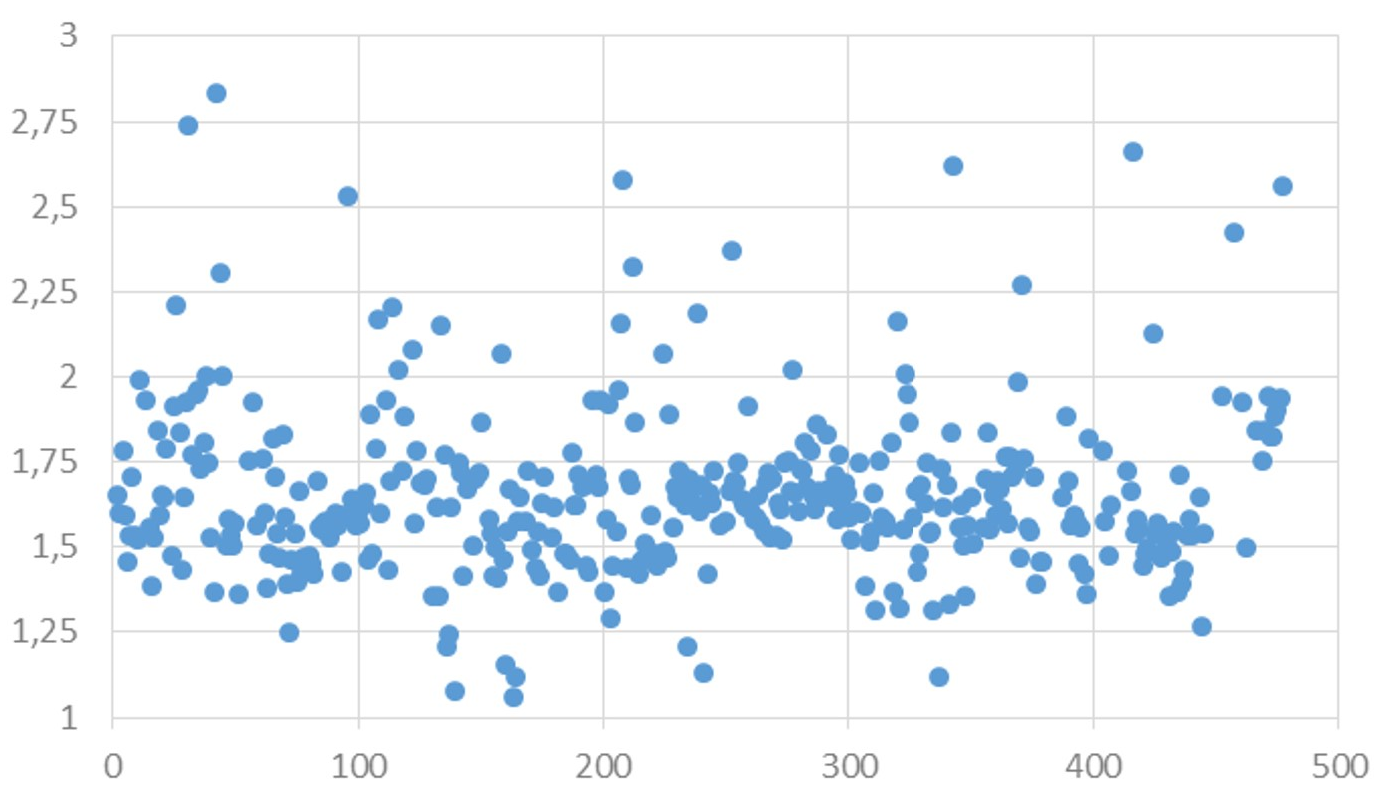

Supplement: Supplementary Figure 2 — Ca/P ratio determined by EDX for all analyses performed. The highest concentration of points corresponds to the range between 1.5 and 1.75, which includes WHL and CDHA (1.5) and HAP (1.67); followed by the region between 1.3 and 1.5, which includes OCP (1.33). [file Image2.tiff]
